# Investigating the causal association between plasma lipids and the risk of squamous cervical cancer: A two-sample mendelian randomization study

**DOI:** 10.5937/jomb0-58020

**Published:** 2025-11-05

**Authors:** Yuemei Cui, Ya Li, Jing Na, Junling Lu, Xinyou Wang, Shichao Hanv, Jun Wang

**Affiliations:** 1 Dalian Medical University, Dalian, Liaoning, 116044, China; 2 Department of Gynecology and Obstetrics, Dalian Municipal Central Hospital, Affiliated of Dalian University of Technology, Dalian 116033, China; 3 Department of Gynecology and Obstetrics, Second Affiliated Hospital of Dalian Medical University, Dalian 116000, China

**Keywords:** Mendelian randomization, squamous cervical cancer, plasma lipids, dyslipidemia, human genetics, Mendelska randomizacija, skvamozni karcinom grlića materice, lipidi u plazmi, dislipidemija, humana genetika

## Abstract

**Background:**

This study aimed to investigate the causal relationship between plasma lipid levels-total cholesterol (TC), triglycerides (TGs), low-density lipoprotein cholesterol (LDL-C), and high-density lipoprotein cholesterol (HDL-C)and the risk of squamous cervical cancer (SCC) using Mendelian Randomization (MR).

**Methods:**

Genome-wide association study (GWAS) data for plasma lipid traits were obtained from the Global Lipids Genetics Consortium (GLGC), and SCC outcome data were sourced from the FinnGen consortium. The primary analysis was conducted using the inverse variance weighted (IVW) method, supported by Mr-Egger regression, weighted median, and weighted mode approaches. Sensitivity analyses were performed to assess the robustness of the results, and the Steiger test was used to evaluate the directionality of the associations.

**Results:**

The IVW analysis revealed that higher plasma levels of TC (OR: 1.777; 95% CI: 1.118-2.825; p = 0.015) and LDL-C (OR: 1.674; 95% CI: 1.013-2.767; p = 0.044) were associated with an increased risk of SCC. No significant associations were found between TGs (OR: 0.644; 95% CI: 0.343-1.212; p = 0.173) or HDL-C (OR: 1.050; 95% CI: 0.616-1.790; p = 0.857) and SCC.

**Conclusions:**

This study provides evidence of a causal relationship between elevated plasma TC and LDL-C levels and a higher risk of SCC, highlighting a novel aspect of SCC etiology. These findings may inform further functional and clinical research in the progression of SCC.

## Abstract (sr)

**Uvod:**

Ova studija je imala za cilj da istraži uzročnu vezu između nivoa lipida u plazmi-ukupnog holesterola (TC), triglicerida (TG), holesterola lipoproteina niske gustine (LDL-C) i holesterola lipoproteina visoke gustine (HDL-C)i rizika od skvamoznog karcinoma (RMSC) i mendelijalnog karcinoma.

**Metode:**

Podaci studije o asocijacijama na nivou genoma (GVAS) za vrednosti lipida u plazmi dobijeni su od Globalnog konzorcijuma za genetiku lipida (GLGC), a podaci o ishodu SCC su dobijeni iz konzorcijuma FinnGen. Primarna analiza je sprovedena korišćenjem metode ponderisane inverzne varijanse (IVV), podržane Mr-Eggerovom regresijom, ponderisanim medijanom i pristupom ponderisanom modu. Urađene su analize osetljivosti da bi se procenila robustnost rezultata, a Stajgerov test je korišćen za procenu usmerenosti asocijacija.

**Rezultati:**

IVV analiza je otkrila da su viši nivoi TC u plazmi (OR: 1,777; 95% CI: 1,118-2,825; p = 0,015) i LDL-C (OR: 1,674; 95% CI: 1,013-2,767; p = 0,044) bili su povezani sa povećanim rizikom od SCC. Nisu pronađene značajne veze između TG (OR: 0,644; 95% CI: 0,343-1,212; p = 0,173) ili HDL-C (OR: 1,050; 95% CI: 0,616-1,790; p = 0,857) i SCC.

**Zaključak:**

Ova studija pruža dokaze o uzročnoj vezi između povišenih nivoa TC i LDL-C u plazmi i većeg rizika od SCC, naglašavajući novi aspekt etiologije SCC. Ovi nalazi mogu dati informaciju daljim funkcionalnim i kliničkim istraživanjima u progresiji SCC.

## Introduction

Cervical cancer (CC) represents a significant global health challenge, ranking as the third most prevalent cancer and the fourth leading cause of cancer-related deaths among women [1]. Despite advancements in screening and vaccination, CC remains a major contributor to cancer morbidity and mortality, especially in low- and middle-income countries. Squamous cervical cancer (SCC) is the predominant type of CC, accounting for approximately 80-90% of all diagnosed cases. SCC has a complex etiology, primarily caused by persistent high-risk human papillomavirus (hr-HPV) infection [2]. However, not all HPV infections progress to cancer, suggesting the involvement of additional cofactors in the carcinogenic process.

Dyslipidemia is characterized by an imbalance in plasma cholesterol and triglyceride levels, including total cholesterol (TC), triglycerides (TGs), low-density lipoprotein cholesterol (LDL-C), and high-density lipoprotein cholesterol (HDL-C). It presents as increased plasma levels of TC, TGs, or LDL-C, decreased HDL-C levels, or a combination of these abnormalities [3]. Dyslipidemia contributes to life-threatening cardiovascular diseases and significantly influences the development and prognosis of various malignant tumors. The pathogenic mechanism of dyslipidemia has not been fully elucidated, potentially linked to inflammation-driven carcinogenesis resulting from abnormal lipid metabolism [4]. Collado et al. [5] observed that dyslipidemia correlates with an elevated recurrence rate of CC, indicating the potential predictive significance of TC and TGs in CC prognosis. In a prospective study, Frontela-Noda et al. [6] found that women with squamous intraepithelial cervical lesions had higher TGs levels than healthy women. These suggest that dyslipidemia may be a significant factor in both the progression of cervical lesions and the increased risk of CC. The relationship between plasma lipids and CC is not yet fully understood, as observational studies are often complicated by potential confounding factors and the possibility of reverse causation.

Mendelian randomization (MR) presents a robust methodology for confronting these challenges, by harnessing genetic variations as instrumental variables (IVs) to deduce causality. MR leverages the random distribution of genes at conception to minimize confounding and reverse causation, providing more accurate estimates of the causal effects of exposures on outcomes [7].

This study utilized MR to assess the causal relationship between genetic variants linked to plasma lipid traits and the risk of SCC, aiming to enhance understanding of SCC causes and identify biomarkers for risk assessment and targeted treatments.

## Materials and methods

### Study design

**Figure 1:**
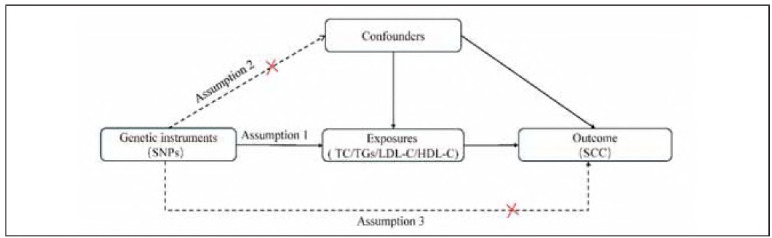
Overview of the current Mendelian randomization (MR) analysis.

This two-sample MR analysis evaluated plasma levels of TC, TGs, LDL-C, and HDL-C as exposure variables and investigated the incidence of SCC as the outcome. Figure 1 presents a schematic of the MR study design. For a robust MR analysis, the following criteria are crucial: (A) The chosen Single Nucleotide Polymorphisms (SNPs) must have a strong association with the exposure; (B) The SNPs should not be influenced by confounding variables; and (C) The SNPs must impact the outcome exclusively through the exposure.

### GWAS data for plasma lipids and SCC

The analysis employed published summary statistics from publicly available GWAS, specifically focusing on European populations and including both male and female individuals (Table 1) plasma lipids. Plasma levels of TC, TGs, LDL-C, and HDL-C were sourced from the Global Lipids Genetics Consortium (GLGC) study, which analyzed the association between these lipid traits and SNPs. The GLGC study conducts a genome-wide genotyping analysis on 188,577 individuals to identify loci linked to plasma lipid levels.

**Table 1:** Details of Genome-Wide Association Studies (GWAS) Included in the Mendelian Randomization Analysis.

Trait	year	Consortium	Population	Sample Size	n Case	n Control	n SNPs	GWAS ID
TC	2013	GLGC	European (~96%),<br>non-European (~4%)	187,365	NA	NA	2,446,982	ieu-a-301
LDL-C	2013	GLGC	European (~96%),<br>non-European (~4%)	173,082	NA	NA	2,437,752	ieu-a-300
TGs	2013	GLGC	European (~96%),<br>non-European (~4%)	177,861	NA	NA	2,439,433	ieu-a-302
HDL-C	2013	GLGC	European (~96%),<br>non-European (~4%)	187,167	NA	NA	2,447,442	ieu-a-299
SCC	2024	FinnGen	European	201,706	212	201,494	20,085,145	NA

Squamous cervical cancer. SNPs linked to SCC were examined using data from the R11 release of genome-wide analysis results in FinnGen, encompassing 201,494 individuals after excluding those with ambiguous gender, high genotype missingness (>5%), excess heterozygosity (±4 SDs), and non-Finnish ancestry. Patients with SCC were identified using ICD-O-3 codes. The FinnGen study is a large-scale genomics project that has examined over 500,000 samples from the Finnish biobank to link genetic variations with health data, with the goal of elucidating disease mechanisms and predispositions. This initiative involves collaboration between Finnish research organizations, biobanks, and international industry partners. Given that the GWAS data are publicly accessible and have been approved by relevant ethical review boards, no additional ethical approval was required for this analysis.

### Instrumental variables selection

We established uniform standards for selecting genetic variants associated with plasma lipids. Initially, SNPs with genome-wide significance (*p* < 5 X 10^-8^) were selected. We set an Linkage Disequilibrium(LD) threshold of R^2^ < 0.001 and a clumping distance of 10,000 kb to select uncorrelated SNPs. We concurrently collected data, including beta values and standard errors for each SNP to compute the F-statistic. SNPs with an F-statistic > 10 were retained to ensure a robust association with the exposure factor and to mitigate inaccuracies due to weakly correlated SNPs. The PhenoScanner tool [8]. was used to cross-reference the IVs and remove SNPs with genome-wide significance related to potential confounders or outcomes. We applied a primary threshold of *p* < 1e-5 and a criterion of *p* < 0.001 to analyze the potential relationship between plasma lipids and SCC. Horizontal pleiotropy in the SNPs was evaluated using the MR-Egger intercept test. A *p* greater than 0.05 in the MR-Egger intercept test indicates no horizontal pleiotropy, suggesting the causal relationship is free from confounding factors. The MR-PRESSO global test was employed to iteratively calculate the *p* for the residual SNPs until it exceeded 0.05. These remaining SNPs were then applied in subsequent MR analyses.

### Two samples of MR analysis

We utilized four methods for two-sample MR analysis: inverse-variance weighted (IVW), MR-Egger regression, weighted median, and weighted mode. Effect estimates were primarily assessed using the IVW method as the principal analytical tool [9]. A *p* below 0.05 signifies a statistically significant causal relationship. Additional checks were conducted to ensure robust and reliable outcomes. For example, when multiple analytical methods consistently indicate the same direction of causality, it reinforces the validity of that causal inference. The Steiger test was employed to ascertain causality direction, with a *p* < 0.05 indicating a unidirectional causal relationship and reducing the risk of reverse causation.

### Sensitivity analysis

**Figure 2:**
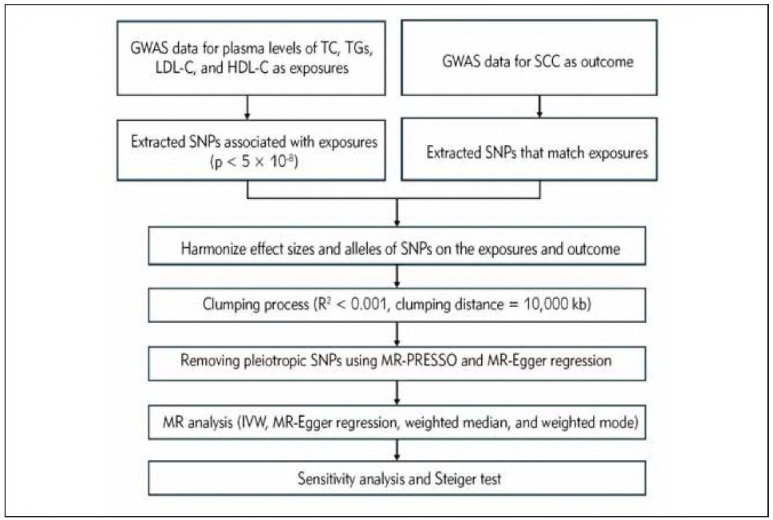
The flowchart of the current Mendelian randomization (MR) analysis.

Sensitivity analyses were performed to validate the robustness of our results. These studies included Cochran's Q test, MR-Egger regression, and leave-one-out analysis. Cochran's Q test evaluated heterogeneity among the IVs, with a *p *< 0.05 indicating significant heterogeneity. Depending on the presence of heterogeneity, either a random-effects or a fixed-effects IVW method was applied. The intercept from the MR-Egger regression served as a reliable indicator of horizontal pleiotropy. A leave-one-out test was conducted to assess the impact of individual SNPs on the causal relationship. All analyses were performed using the TwoSampleMR (version 0.5.7), Mendelian-Randomization (version 0.8.0),and MRPRESSO package (1.0) in R Software 4.3.1 (https://www.R-project.org). Figure 2 depicts the flowchart for the MR analysis process.

## Results

### SNP selection

After LD clumping and excluding potential confounders, we identified independent SNPs associated with plasma lipids. These variables exhibited strong statistical power, indicated by F statistics greater than 10.

### Causality estimates between plasma levels of TC, TGs, LDL-C, HDL-C, and SCC Risk

**Figure 3:**
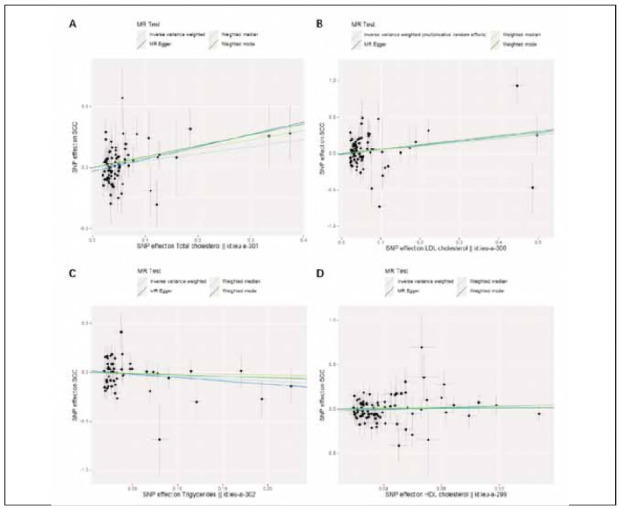
The causality of plasma lipid levels on the risk of SCC. (A) Comparing MR results for the relationship between TC levels and SCC. (B) Comparing MR results for the relationship between LDL-C levels and SCC. (C) Comparing MR results for the relationship between TGs levels and SCC. (D) Comparing MR results for the relationship between HDL-C levels and SCC.

Figure 3 shows that the IVW analysis identified a significant association between elevated plasma levels of TC (OR, 1.777; 95% CI, 1.118-2.825; *p* = 0.015) and LDL-C (OR, 1.674; 95% CI, 1.0132.767; *p* = 0.044) with a higher risk of SCC. Genetically predicted levels of TGs (OR, 0.644; 95% CI, 0.343-1.212; *p* = 0.173) and HDL-C (OR, 1.050; 95% CI, 0.616-1.790; *p* = 0.857) are not associated with SCC risk (see Figure 3).

**Figure 4:**
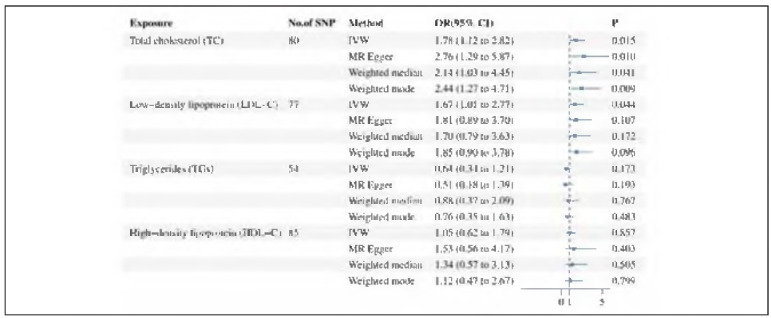
Forest plot for the causality of plasma lipid levels on SCC derived from MR analysis. CI, confidence interval; IVW, inverse-variance weighted; OR, odds ratio; SNPs, single nucleotide polymorphisms.

The consistency of these associations across the three additional MR analysis methods underscores the robustness of the primary results. An overview of the MR estimates is provided in Figure 4.

Although some methods had broad 95% confidence intervals, the MR-Egger intercept test and Cochran's Q test showed no evidence of pleiotropy or heterogeneity among SNPs for TC, TGs, and HDL-C (Table 2).

**Table 2:** Tests of pleiotropy of selected SNPs and heterogeneity between SNPs.

Exposure	Horizontal pleiotropy		Heterogeneity (MR Egger test)	
	Beta(SE)	*P*	Cochran's Q	*P*
TC	0.022	0.154	76.254	0.535
LDL-C	0.024	0.761	112.635	0.003
TGs	0.026	0.551	57.871	0.268
HDL-C	0.023	0.381	79.726	0.581

Due to moderate heterogeneity in LDL-C instrumental variables (Cochran's Q test, *I* ^2^ = 32.6%), the IVW method with random effects was used to address this issue. Moreover, the MR-PRESSO global test did not detect any pleiotropic effects (*p* > 0.05). The Steiger test indicated that the variance explained in the exposure significantly exceeded that in the outcome. The leave-one-out analysis verified that no individual SNP had a significant impact on causality.

## Discussion

This study represents the first MR analysis exploring the causal relationship between plasma lipids and SCC. Our findings indicate that elevated TC and LDL-C levels are associated with an increased risk of SCC, while TGs and HDL-C show no significant association. These results highlight TC and LDL-C as potential contributors to SCC pathogenesis, offering novel insights into metabolic mechanisms in cervical carcinogenesis.

Cervical cancer, predominantly SCC, remains a major global health burden, driven primarily by persistent hr-HPV infection. However, the incomplete penetrance of HPV underscores the role of cofactors, including dyslipidemia [2]. Cholesterol, a key component of lipid rafts, interacts with cellular membranes and signaling pathways, influencing processes like proliferation and inflammation [10]. While prior studies on TC and SCC yield conflicting results-some reporting positive correlations and others inverse associations [11]-our MR analysis aligns with Yu et al. [12] who identified higher TC levels in SCC patients versus controls. Mechanistically, elevated TC may promote carcinogenic signaling via cholesterol-rich lipid rafts, activating pathways linked to atypical cell proliferation [5]. Additionally, hypercholesterolemia correlates with inflammatory markers (e.g., IL-6, IL-8), suggesting inflammation-mediated oncogenesis. Targeting cholesterol regulation could thus offer therapeutic avenues for SCC management.

LDL-C, the primary cholesterol transporter to peripheral tissues, may fuel tumor growth by supplying biosynthetic substrates. Our findings align with clinical observations of elevated LDL-C in well-differentiated SCC and advanced CC stages. Oxidized LDL (Ox-LDL) further stimulates cancer progression in other malignancies [13], implying a plausible role in SCC. However, heterogeneity in LDL-C instrumental variables and discrepancies across MR methods (e.g., non-significant results in MR-Egger) warrant caution. Residual pleiotropy or methodological limitations may underlie these inconsistencies, necessitating further mechanistic studies.

Contrary to prior reports, our analysis found no association between TGs/HDL-C and SCC risk. While TGs are implicated in energy provision and membrane synthesis, and HDL-C exhibits anti-inflammatory properties linked to cancer prevention [14], conflicting evidence exists. Sun et al. [15] similarly reported no dyslipidemia-CC association in Chinese cohorts. Lifestyle factors and metabolic heterogeneity may obscure these relationships, emphasizing the need for molecular studies on lipid metabolism enzymes and receptors in SCC.

As the first two-sample MR study on this topic, our approach minimizes confounding and reverse causation, bolstered by sensitivity analyses and the Steiger test. The identification of TC and LDL-C as risk factors underscores their potential clinical relevance. However, limitations include the European ancestry of participants, limiting generalizability, and unresolved pleiotropy risks inherent to MR. Future research should validate these findings in diverse populations and elucidate biological mechanisms linking lipid profiles to SCC.

## Conclusion

Our research provides critical insights into the relationships between specific plasma lipid profiles and the risk of developing SCC. Our findings indicate that higher TC and LDL-C levels are linked to a greater risk of SCC. In contrast, TGs and HDL-C appear to have no significant impact on the development of SCC. These findings improve our comprehension of the impact of lipid profiles on SCC risk and highlight the necessity for additional research to clarify the underlying mechanisms.

## Dodatak

### Acknowledgments

The authors express their gratitude to the individuals who participated in all the GWAS cohorts involved in this research, as well as to the researchers of the IEU Open GWAS project. We wish to acknowledge the participants and investigators of the FinnGen study for their collaboration in providing the GWAS summary statistics.

### Financial support

This work was supported by the Liaoning Province Applied Basic Research Program Project (2023JH2/101300068).

### Contributors

Yuemei Cui: conceived the study and was the major contributor to the research and the writing of the manuscript; Ya Li, Jing Na, and Sichao Han: summarized the literature, typeset the articles, and embellished the language; Junling Lu and Xinyou Wang: conducted data analysis; Jun Wang: revised the manuscript and provided supervision.

### Conflict of interest statement

All the authors declare that they have no conflict of interest in this work.
